# Behavioral and neurobiological implications of kairomones for rodents: an updated review

**DOI:** 10.3389/fnins.2025.1485312

**Published:** 2025-02-19

**Authors:** Diya Manjunath, Hayavadhan Sampath, Roy N. Kirkwood, Sinsha Santhosh, Devaraj Sankarganesh

**Affiliations:** ^1^School of Bio Sciences and Technology, Vellore Institute of Technology, Vellore, Tamilnadu, India; ^2^School of Animal and Veterinary Sciences, The University of Adelaide, Roseworthy, SA, Australia

**Keywords:** house mouse, rat, rodent control, pest management, odorants, pheromones, sulfates

## Abstract

Like many other vertebrates, rodents communicate via pheromones, which favors intraspecies communication. In contrast, kairomones are semiochemicals involved in interspecific communication, facilitating information between organisms of different species but advantageous for the receiver. Kairomones induce behavioral, physiological, and endocrinological changes in rodents, and have been proven to activate specific neuronal pathways in one or multiple components of the olfactory system (the main olfactory system, accessory olfactory system, and Gruenberg ganglion). The sophisticated olfactory networks help rodents innately recognize kairomones and elicit appropriate behavioral (aversive, avoidance, defense, and escape mechanisms), physiological, and endocrinological changes. Thus far, odor sources (e.g., urine, feces, hair, and body rubbings) of predators, such as felines, canines, and serpentes, have been studied in rodents. Specific kairomones have been identified, behaviorally tested, and validated for their potential to induce behavioral, neuronal, and endocrinological changes in rodents. One of the most studied kairomones is the fox odor, 2,5-dihydro 2,4,5-trimethylthiazoline, although other compounds have been reported to a limited extent. This review summarizes the current knowledge on kairomones and their effects on the behavioral, neuronal, and endocrine systems of rats and mice.

## Introduction

Chemical signals are pivotal in the social communication between rodents and may impact their reproduction and survival. Pheromones are intra-species chemical signals released by one individual of the species that elicit definite neuroendocrinological changes in another individual of the same species. In nature, pheromones are secreted by either males or females and elicit the responses of their conspecifics. Pheromones are present in the urine, feces, saliva, cervical mucus, tears, and glandular secretions of rodents, and they have been extensively studied (Tirindelli et al., 2009). However, the related chemical signals, kairomones, have not received the same attention. Kairomones are chemical signals released by one organism that elicit a behavioral response in another organism of a different species and are advantageous to the receiver (Brown et al., 1970). Kairomones help the receiver in detecting and avoiding predators (Muller-Schwarze, 2006; Rajchard, 2013). This ability to detect and respond to kairomones can be critical for the survival and reproduction of the receiver species, as it allows them to exploit resources or evade threats (Tirindelli et al., 2009).

**Figure 1:**
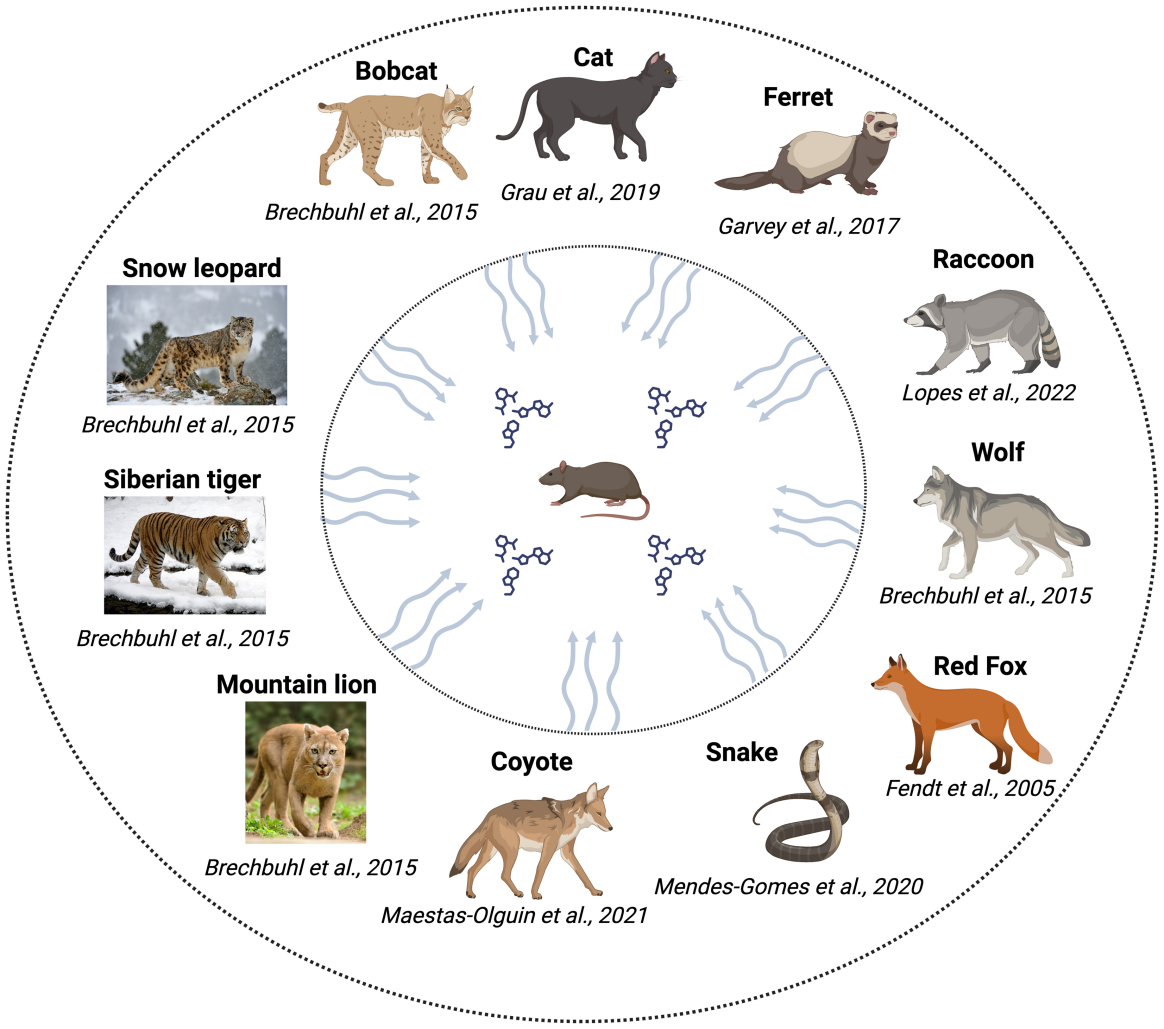
Predators investigated for the identification of kairomones/testing of kairomone sources with rodents.

Kairomones include volatile, semi-volatile, and non-volatile organic compounds. However, kairomones are secreted into limited body fluids, such as urine, feces, and glandular secretions, and the composition of each type of secretion varies significantly (Fortes-Marco et al., 2013). Other sources, such as the fur of cats (May et al., 2012) and the skin of snakes (Papes et al., 2010), have also been reported as sources of kairomones. The various predators that have been shown to serve as sources of kairomones are shown in Figure 1. All the listed animals in the figure have been shown to be predators of rodents, excrete kairomones and are implicated in physiological/ behavioral/ endocrinological changes in rodents. Although evidence is available for rats being a predator of mice, we have not focused on this aspect in this review.

Mammalian kairomones, particularly rodent kairomones, are secreted by other species and detected by the rodents, and are mostly nitrogen-containing amine compounds resulting from the specific metabolism of carnivores (Moine et al., 2018). Rodents have sophisticated olfactory systems that receive and process various stimuli. The highly organized olfactory systems and subsystems help rodents process both favorable and threatening stimuli through which they coordinate complex physiology and thereby survive (Liberles, 2014, 2015). Kairomones thus serve as threat stimuli and provide information about the predator and indicate the presence of the predator to the prey.

To shed light on the role of kairomones in behavioral and neurobiological effects, in this review, we focus on kairomones and their experimental evidence with common rodents (mice and rats). We aimed to provide a detailed perspective on kairomones to demonstrate the current knowledge gaps that could be addressed to develop this fascinating field and to conduct translational research to develop applications/formulations for rodent repellents.

## Rodent behavioral response to kairomones

Behavioral expressions in rodents are complex and coordinated in response to stimuli. For example, sexual stimuli activate approach behaviors (Le Moene and Agmo, 2018), whereas kairomones elicit aversive behaviors and this aversion is dose-dependent (Vasudevan and Vyas, 2013). Thus, the complexity of behavior is due to the highly organized neural system that coordinates other physiological events in response to stimuli (Kingsbury et al., 2019). For instance, defense (flight behavior and lunge-and-bite attacks on opponents) is an escape mechanism exhibited by the rodents (Adams, 1980). Risk assessment behavior (stereotypical low-lying body posture) is also a type of defensive behavior in rodents (Blanchard and Blanchard, 1989). It involves a series of actions, such as verification, recognition, and spatial orientation of the threat. Avoidance is also a characteristic behavior, evidenced by withdrawal from the injurious or dangerous stimuli (Wernecke and Fendt, 2015). Brechbühl et al. (2015) measured the avoidance behavior in mice using elevated blood pressure and heart rates. Freezing is another behavior, which depends on the environmental conditions, wherein the animals explore the possibility for escaping or hiding, and remain immobile except for breathing (Taugher et al., 2015). Freezing is different from tonic immobility, which is accompanied by reduced motion, and is activated at intermediate levels of predator threat (Roelofs, 2017). This freezing behavior was noticed in rats when exposed to 2,4,5-Trimethylthiazoline (Fortes-Marco et al., 2015).

Behavioral and physiological changes of the prey also differ following acute or chronic exposure to predator odors. In acute exposure, the prey exhibit decreased activity and non-defensive behaviors (feeding, drinking, etc.), and location change by hiding and /or flight are being the most common. However, in the case of chronic exposure, sensitization and habituation are the two main realms intricately involved in the expression of complex behaviors by changing physiological, behavioral, and neuroendocrinological parameters (Hegab et al., 2015). However, a clear understanding of kairomone-associated behaviors is required before suggesting a compound is a kairomone.

## Kairomones: sources and compounds

As described in more detail below, various studies have analyzed different sources of kairomones and reported potential compounds, with a few demonstrating the behavioral, endocrinological, and neuronal effects of these compounds (e.g., TMT), confirming their kairomone properties. In addition, the effects of cues from fur and feces (Grau et al., 2019), and body odor (Garvey et al., 2017; Masini et al., 2010) of ferrets, the urine of wolves (Osada et al., 2013), and wild rattlesnakes (Mendes-Gomes et al., 2020) on rodents were also noted. The most prominent sources were secretions from cats, foxes, ferrets, coyotes, and other related species. Molecules such as Feld1 and Feld4 (proteins), L-feline (amino acid derivative), 2,5-dihydro-2,4,5-trimethylthiazoline (TMT) (thiazoline compound), and 2-phenylethylamine (amine compound) have been the most tested.

### Feline kairomones and trimethylthiazoline (TMT)

Cat collars were shown to induce fear behaviors, autonomic responses (unconditioned and conditioned), and endocrinological and olfactory changes in rodents, suggesting they are a potential source of kairomones (Dielenberg et al., 2001a, 2001b; McGregor et al., 2002, 2004; Staples et al., 2008b). May et al. (2012) tested fur, a collar worn by cats, and a cloth rubbed on cats, and attributed kairomone properties to the collar and rubbed cloth. Cat urine produced a repellent effect in rats, suggesting that it is a source of kairomones (Mulungu et al., 2016). Cat urine revealed the presence of felinine, a urinary molecule excreted in a sex-and age-dependent manner (Miyazaki et al., 2006). Felinine blocked pregnancy in female rats and decreased testosterone levels in male rats, rendering it a dual-purpose molecule (pheromone in cats and kairomone in rats) (Voznessenskaya, 2014; Voznessenskaya and Laktionova, 2019). Felinine is a sulfur-containing amino acid and, therefore, it would be pertinent to search for felinine-like molecules (and other amino acids) in the urine of cats to identify potentially novel kairomones. A few studies have identified protein molecules in cats as kairomones. Papes et al. (2010) demonstrated that Feld 4, a cat allergen, was a kairomone and evidenced behavioral (avoidance and risk assessment behaviors), endocrinological (release of ACTH), and olfactory effects (TrpC2-mediated vomeronasal organ (VNO) processing). In contrast, Feld1, a cat fur protein, did not evoke significant fear responses in rodents, indicating that not all cat fur proteins are kairomones (Grau et al., 2021).

Although cat odor sources were efficient in inducing multiple-level effects in rodents, putative compounds from many sources have not yet been identified. For instance, cat urine and fur have been shown to elicit fear behaviors in rodents. Nevertheless, the only identified kairomones from cats are felinine and Feld1. However, given the various biological effects of these sources, they may contain volatile, semi-volatile, and/or non-volatile compounds that need to be identified in order to search for more promising molecules as kairomones. Miyazaki et al. (2006) identified four derivatives of felinine (3-mercapto-3-methyl-1-butanol, 3-mercapto-3-methylbutyl formate, 3-methyl-3-methylthio-1-butanol, and 3-methyl-3-(2-methyldisulfanyl)-1-butanol) in cat urine, however, their separate kairomonal properties have yet to be tested. In addition, the mammary area of cats contains appeasing pheromones (a mixture of fatty acids, such as linoleic, oleic and palmitic acids) (Mills et al., 2013; Pageat, 1999) that have yet to be validated for kairomone effect.

TMT, originally identified in fox feces, induced fear in laboratory rodents (Vernet-Maury et al., 1977, 1984). Since then, various forms of TMTs (2,5-dihydro-2,4,5-trimethylthiazoline/ 2,4,5-trimethyl thiazoline/ Trimethylthiazoline/ 2,3,5-Trimethyl-3-thiazoline) have been implicated in the modulation of behavior (innate, stress, fear, and anxiety), endocrine systems, and neuronal/olfactory systems and subsystems in rodents (Morrow et al., 2000; Hebb et al., 2002; Fendt et al., 2005; Fendt and Endres, 2008; Rosen et al., 2008; Janitzky et al., 2009; Hacquemand et al., 2010). These studies have proven that TMT is an effective kairomone. However, Rampin et al. (2018) did not find TMT in fox feces, suggesting its’ presence to be inconsistent. We suggest using efficient extraction techniques (e.g., solid-phase microextraction and stir-bar sorptive extraction) to identify more potent molecules in fox feces and to test their kairomonal properties by conducting appropriate behavioral studies in rodents. Taken together, the identification and quantification of TMT in fox feces and determining which TMT is an effective kairomone remain open questions.

### Other kairomone sources and compounds

Urine samples from various carnivores (foxes, bobcats, pumas, and coyotes) induced high avoidance behaviors in rodents, suggesting that they are potential sources of kairomones (Wernecke and Fendt, 2015). The puma urine induced high blood pressure in mice, advocating it as a kairomone source (Brechbühl et al., 2015). Perez-Gomez et al. (2015) observed innate aversion in mice toward four kairomone sources or compounds; TMT, cat fur odor, 2-phenylethlamine (a molecule in coyote urine), and 2-propylthietane (a molecule in stoat urine). In another study, bobcat urine induced high avoidance behaviors in rats (St-Cyr and McGowan, 2015). In addition, coyote urine and 2-phenylethylamine have also been found to possess kairomone properties (Pentkowski et al., 2022; Maestas-Olguin et al., 2021). The urine of pumas and raccoons, and the anal gland secretions of skunks, induced a high immobility index in rodents (Lopes et al., 2022), supporting the presence of potent kairomones in these sources. Wolf urine induced avoidance and freezing behavior in mice, wherein the compounds in the urine samples were identified as pyrazines (2,6-dimethylpyrazine (DMP), trimethylpyrazine (TMP), and 3-ethyl-2,5-dimethyl pyrazine (EDMP)) (Osada et al., 2013, 2015). The urine, fur, and body odor of ferrets were also tested with rodents but only in the context of behavioral modification. The effect of these odors on the physiological and neuronal systems has not been evaluated and the compounds in these sources were not identified in these studies (Grau et al., 2019; Garvey et al., 2017). Though wild rattlesnakes were directly tested with rat behavior and neuronal activation using c-fos, the responsible compounds have not been identified (Mendes-Gomes et al., 2020).

Various kairomones (compounds/sources) tested with rodent behavior are listed in Table 1, which indicates that TMT is the most widely tested kairomone compound and that the putative kairomone sources of many predators have not yet been tested and warrant further investigation. We infer from the above studies that choosing the kairomone source is crucial because rodents may assess the threat level of the predator variably, such as being most, moderate, or least threatening. A comparative analysis of various kairomones and the availability of behavioral and/or neuronal evidence are depicted in Table 2.

**Table 1:** Behavioral effects of various kairomones tested with different species and strains of rodents.

Kairomone compound/kairomone source	Test animals	Behaviors tested/observed	Reference
2,5-Dihydro-2,4,5-trimethylthiazoline	Adult naïve female OF1 mice	Anxiety-like behaviors and fear-like behaviors	Hacquemand et al. (2013)
2,5-Dihydro-2,4,5-trimethylthiazoline + other molecules	C57BL/6JRj mice	Exploratory, avoidance, anti-predatory, and fear behaviors	Grau et al. (2021)
	Sprague-Dawley rats	Fear responses	Hotsenpiller and Williams (1997)
	Brown Norway and Wistar rats	Exploration, freezing, pushing litter, grooming, resting, and position change	Rampin et al. (2018)
Trimethylthiazoline	Naïve Long-Evans hooded rats	Avoidance and freezing behaviors	Blanchard et al. (2003)
	Sprague–Dawley rats.	Freezing and fear responses	Rosen et al. (2008)
2,4,5- trimethylthiazoline	Adult OF-1 mice	Immobility, freezing, velocity and distance to odorant	Buron et al. (2007)
2,4,5-Trimethylthiazoline and Toluene	Naïve OF-1 mice	Preference and avoidance test; freezing and velocity in circular open field	Hacquemand et al. (2010)
2-phenylethylamine	Long-Evans hooded rats	Number of transits, locomotor activity, avoidance, freezing, contact, risk assessment, rearing, and sniffing,	Maestas-Olguin et al. (2021)
	Adult Sprague–Dawley rats	Avoidance behaviors	Ferrero et al. (2011)
Fox urine	Naïve Sprague-Dawley rats	Fear behaviors (locomotor activity and avoidance behavior)	Wernecke et al. (2015)
Collars worn by cats, cloth rubbed over a cat, or cat fur	Albino Wistar rats and female Sprague-Dawley rats	Food consumption, latency to reach the food, and total distance travelled	May et al. (2012)
Cat collar	Wistar rats	Stimulus contact, grooming and rearing	McGregor et al. (2004)
Urine of mountain lion and raccoon; Urine and anal gland secretions from the skunk	OMP-GFP mice	Walking distance index and immobility index	Lopes et al. (2022)
Neck swab of cats, shed skin of snake, and urine of rats	Inbred C57BL/6J mice	Avoidance and risk assessment behavior	Papes et al. (2010)
Ferret fur and feces, snake sheds, fox feces, dog feces, and cat urine	Swiss albino mice	Avoidance behaviors	Grau et al. (2019)
Body odor of ferrets	Ship rats (*Rattus rattus*).	Site occupancy and additional behaviors	Garvey et al. (2017)
Body odor of ferrets	Sprague Dawley rats	Defensive behaviors and plasma corticosterone responses	Masini et al. (2010)
2,6-dimethylpyrazine (DMP), trimethylpyrazine (TMP), and 3-ethyl-2,5-dimethyl pyrazine (EDMP)	C57BL/6J mice	Avoidance and freezing behaviors	Osada et al. (2013)
Brazilian rattler snake	Long-Evans laboratory rats	Freezing, sniffing, stretch attend/approach, and grooming behaviors	Mendes-Gomes et al. (2020)

**Table 2:** Comparative table of compounds/source of kairomones tested with various rodents.

Kairomone compound/sources tested	Test animals	Behavioral evidence available	Neuronal evidence available
2,5-Dihydro-2,4,5 trimethylthiazoline	Adult naïve female OF1 mice	**✓**	**X**
	Male Sprague-Dawley rats	**✓**	**✓**
2,5-Dihydro-2,4,5-trimethylthiazoline + other compounds	Male Sprague-Dawley rats	**✓**	**X**
	Brown Norway and Wistar rats	**✓**	**X**
	C57BL/6JRj mice	**✓**	**X**
	Naïve wild-type male mice (C57BL/6)	**✓**	**✓**
Trimethylthiazoline	Naïve male Long-Evans hooded rats	**✓**	**X**
	Male Sprague-Dawley rats	**✓**	**✓**
	Australian Albino Wistar and Sprague-Dawley rats	**✓**	**X**
	Australian Albino Wistar rats	**✓**	**✓**
2,4,5- trimethylthiazoline	Adult OF-1 mice	**✓**	**X**
2,4,5- trimethylthiazoline with other compounds	Adult OF-1 mice	**✓**	**X**
2,3,5-Trimethyl-3-thiazolin	Adult C57BL/6 mice	**✓**	**✓**
2-phenylethylamine	Male Long-Evans hooded rats	**✓**	**X**
	Adult Sprague– Dawley rats	**✓**	**X**
Fox urine	Naïve male Sprague-Dawley rats	**✓**	**✓**
Urine of mountain lion and raccoon; Urine and anal gland secretions from the skunk	OMP-GFP mice	**✓**	**✓**
Feld4	Inbred C57BL/6J mice	**✓**	**✓**
Feld1a + Trimethylthiazoline	C57BL/6JRj mice	**✓**	**X**
Cat collar	Male Wistar rats	**✓**	**✓**
2-phenylethylamine + other compounds	Ship rats & Polynesian rats	**✓**	**X**
coyote urine and 2- phenylethylamine	Adult male Long- Evans hooded rats	**✓**	**X**
Pyrazines	C57BL/6J mice	**✓**	**✓**
Brazilian rattler snakes	Long-Evans laboratory rats	**✓**	**✓**
Ferret body odor	Ship rats and Swiss Albino mice	**✓**	**X**

**✓ indicates ‘yes’ and X indicates ‘no’.**

## The variables of receiver and their responses

### Strain differences, and physiological and developmental conditions

The complexity of the chemical nature, synthesis rate, and excretion level of kairomones is largely dependent on the various characteristics of the predator. Similarly, it is reasonable to expect differences between the different recipient animal strains when these kairomones are perceived. Lister-Hooded and Warsaw Wild Captive Pisula Stryjek (WWCPS) rats showed differences in freezing behavior toward TMT, with increased corticosterone only in WWCPS rats (Storsberg et al., 2018). Given the above, it is expected that differences could be exhibited at the species level (mice, rats, and other rodents) toward the same kairomone/odor source. If evident, it is essential to relate the test to the desired animal species. For example, developing a repellent for house rats requires the testing of house rats with appropriate kairomones. In addition, the sensitivity to the kairomone must be tested to identify its effective concentration.

St-Cyr et al. (2018) observed an endocrine modulatory effect in the offspring of mice exposed to urine from various predators. Furthermore, during estrus and pro-estrus, rats exhibited fewer defensive behaviors and increased risk assessment behaviors than during metestrus and diestrus (Pentkowski et al., 2018), possibly indicating sex drive can modulate kairomone responses. Variations in sex hormones, together with stress hormones, may differentially regulate the endocrine system and influence behavior in females. We suggest that the exposure to kairomones could result in long-term changes in the reproductive efficiency of females (e.g., fertility rate and oocyte maturation) that warrants experimental evidence. It would be interesting to test the behavior of dams (in the presence of their pups) by keeping kairomones inside the cage. This study would highlight the role of maternal stress and vigilance toward threat stimuli in dams.

### Concentration of the compounds and dimension of the test area

The concentration of kairomones determines the extent of behavioral expression in rodents. Therefore, it is essential to determine the lower threshold concentration for the kairomones. In this regard, Laska et al. (2005) evaluated different concentrations of TMT and found that rats discriminated between concentrations of 0.04 and 0.10 parts per trillion (ppt). Similarly, CD1 mice discriminated between the kairomones of six different predators below 0.1 ppm (Sarrafchi et al., 2013). In mice, 1% (10,000 ppm) and 0.1% (1,000 ppm) TMT induced behavioral changes, whereas 0.01% (100 ppm) TMT did not (Hacquemand et al., 2013). The different percentages of TMT were prepared by dissolving TMT in deionized water, and 10 microliters of each of the concentration was presented to the mice. We speculate that the detection of kairomones by prey should work even at suboptimal concentrations to ensure their survival. It is also possible that at very high concentrations, kairomones may smell like general aversive compounds. Therefore, rodents may not elicit kairomone-specific behavioral and neuronal changes. Above all, validation of sensitivity by expression studies of olfactory components is necessary for verifying concentration-based effects. However, current studies are limited in their evaluation of the concentration-based effect of kairomones on rodents.

The dimensions of the test area are critical for the induction of behavioral or neuronal changes by kairomones. Often, when a high concentration of a compound is tested in a large area, the effect produced is equivalent to the effect produced by a low concentration in a small area. In addition, the valence and chemical nature of compounds determine the behavioral expression in the receiver. For instance, highly volatile molecules can reach the receiver promptly, whereas semi-volatile and non-volatile molecules can provide persistent signals. In this way, TMT activated the medial prefrontal cortices of the amygdala in rats when tested in a small open field but failed to induce the same effect in a large open field test (Morrow et al., 2002).

### Sexual dimorphism

The investigatory and grooming behaviors of female mice were lower than those of males when exposed to the urine of domestic cats and L-felinine (Voznessenskaya et al., 2016), implying a sexual dimorphism in the exhibition of behaviors towards predator odors. Exposure of male and female neonatal Sprague–Dawley rats to ferret bedding material led to the suppression of social behaviors in both sexes, while corticosterone levels increased only in males, rendering support for neonatal sexual dimorphism restricted to endocrine action (Stockman and McCarthy, 2017). However, this study used maternal bedding material as a control, which would perhaps provide an opposite response to kairomones, without any neutral responses. Notably, adult male and female Wistar rats showed no differences in behavior or corticosterone levels in response to TMT (Homiack et al., 2017), implying no pronounced sexual dimorphism in adults.

We infer that rodents perceive kairomone/threat stimuli and categorize them into multiple levels, namely low, moderate, and high. Low level threats may be defined as stimuli that are infrequently encountered or originate from smaller predators, eliciting minimal avoidance behavior. Moderate level threats may be defined as stimuli from larger predators and their kairomones, resulting in moderate avoidance behaviors and physiological stress responses. High level threats may be defined as stimuli from highly dangerous and frequently encountered predators, leading to intense fear responses and significant avoidance behavior. Categorization may depend on various factors, including the size and nature of the predator, the nature of the source of origin (e.g., urine or feces), and the age of the odor. Ultimately, this categorization may help rodents exhibit appropriate behavioral and/or physiological actions. This could explain why none of the kairomone/predator odors induced the same behavioral/physiological effects in males and females. This could also be explained in a comprehensive way by comparing various kairomone sources in a Y-maze behavior assay together with other physiological and endocrinological parameters.

## Neuronal effects of kairomones/sources

### Olfactory systems and subsystems

In the periphery of the olfactory system, the existence of different receptor families facilitates detection of various chemical cues and transduces them into neural signals (Trimmer and Mainland, 2017). The olfactory receptor neurons present in the olfactory epithelium receive the incoming sensory inputs via the olfactory nerve and deliver it to the main olfactory bulb that subsequently reaches the olfactory cortex. All of these parts constitute the main olfactory system (Huilgol and Tole, 2016). The processing of the odor identity and valence are based on the higher order olfactory areas, including piriform cortex. Eventually, the brain transforms the complex stimulus into a neural code and helps identify the odorants and their related attributes (Giessel and Datta, 2014).

Kobayakawa et al. (2007) evidenced that fear responses were induced through different olfactory receptor neurons (ORNs) found in the MOS. Strong fear responses in rodents were caused by the activation of multiple glomeruli in the main olfactory bulbs (MOB) (Saito et al., 2017). Olfactory bulbs have downstream projection neurons and contain mitral and tufted cells that further activate other brain areas (Smith and Bhatnagar, 2019). Sensory neurons project their axons to the olfactory bulb and exhibit control over various regions of the brain (Valverde et al., 1992). The selection of new olfactory bulb neurons is based on the odor identity received from peripheral inputs. These new neurons also receive centrifugal inputs from the olfactory cortex (OC) (Yamaguchi, 2017). The nasal cavity contains the nasal vestibular and olfactory epithelia (OE), which both receive and process kairomone signals (Fortes-Marco et al., 2013).

In addition, trace amine-associated receptor (TAAR) expression is found in the olfactory system and displays the characteristic hallmark of OR (olfactory receptors) expression (Liberles, 2015; Dewan, 2021), implying the possible involvement of the MOS in kairomone detection. Indeed, amines found in the urine of different predators, e.g., phenylethylamine and trimethylamine, activate TAARs (Gainetdinov et al., 2018). For instance, 2-phenylethylamine, which is found in the urine of lions and bobcats, activates TAAR4 (Ferrero et al., 2011). Likewise, ORs such as Olfr20, Olfr30, Olfr57, Olfr376, and Olfr491, respond to TMT and are located at the dorsal olfactory epithelium (Jiang et al., 2015), suggesting the involvement of the MOS in kairomone signaling. Many studies have suggested that the MOS mediates the signaling of airborne chemicals and is less connected to neurohormonal pathways. However, a recent review highlighted that corticotrophin-releasing hormone neurons found upstream of the olfactory cortex responded to predator odors, attesting to stress hormone responses (Shin et al., 2023). Nevertheless, further studies are warranted to validate the neuroendocrine changes associated with TAARs-mediated kairomone signaling. In addition, testing of other kairomones, particularly cat odors, with TAARs is warranted. The cat odor may also contain amine compounds, however, a detailed chemical investigation together with neuronal implications provides more insight.

In addition to the main olfactory system (MOS), rodents also possess an accessory olfactory system (epithelia containing sensory neurons of the vomeronasal or accessory olfactory system), Grueneberg ganglion (GG), and septal organ (SO) (Ennis et al., 2015).

### Accessory olfactory system and Grueneberg ganglion (GG)

C-fos has been recognized as a marker of neuronal activity in rodents (Bullitt, 1990). Cat-odor-induced c-fos expression in accessory olfactory bulb (AOB) mitral cell layers indicates the involvement of the AOB in kairomone signaling (McGregor et al., 2004; Staples et al., 2008a). In addition, calcium influx in vomeronasal sensory neurons (VSNs) and early c-fos expression in the VNO epithelium attest to the role of VNO neurons in kairomone signaling. Specifically, transient receptor potential channel 2 (TrpC2) in VNO neurons is required for cat odor signaling in rodents (Papes et al., 2010). Indeed, TrpC2 is essential in social behaviors and making instinctive decisions in rodents (Zufall, 2014). Together, these studies provide direct evidence that cat odors activate the VNO and accessory components, while rat and snake odors serve as kairomones for mice and induce activation of TrpC2 channels in the VNO. However, the activation of VNO or its related components has not yet been investigated for TMT and other potential amine kairomones.

**Figure 2:**
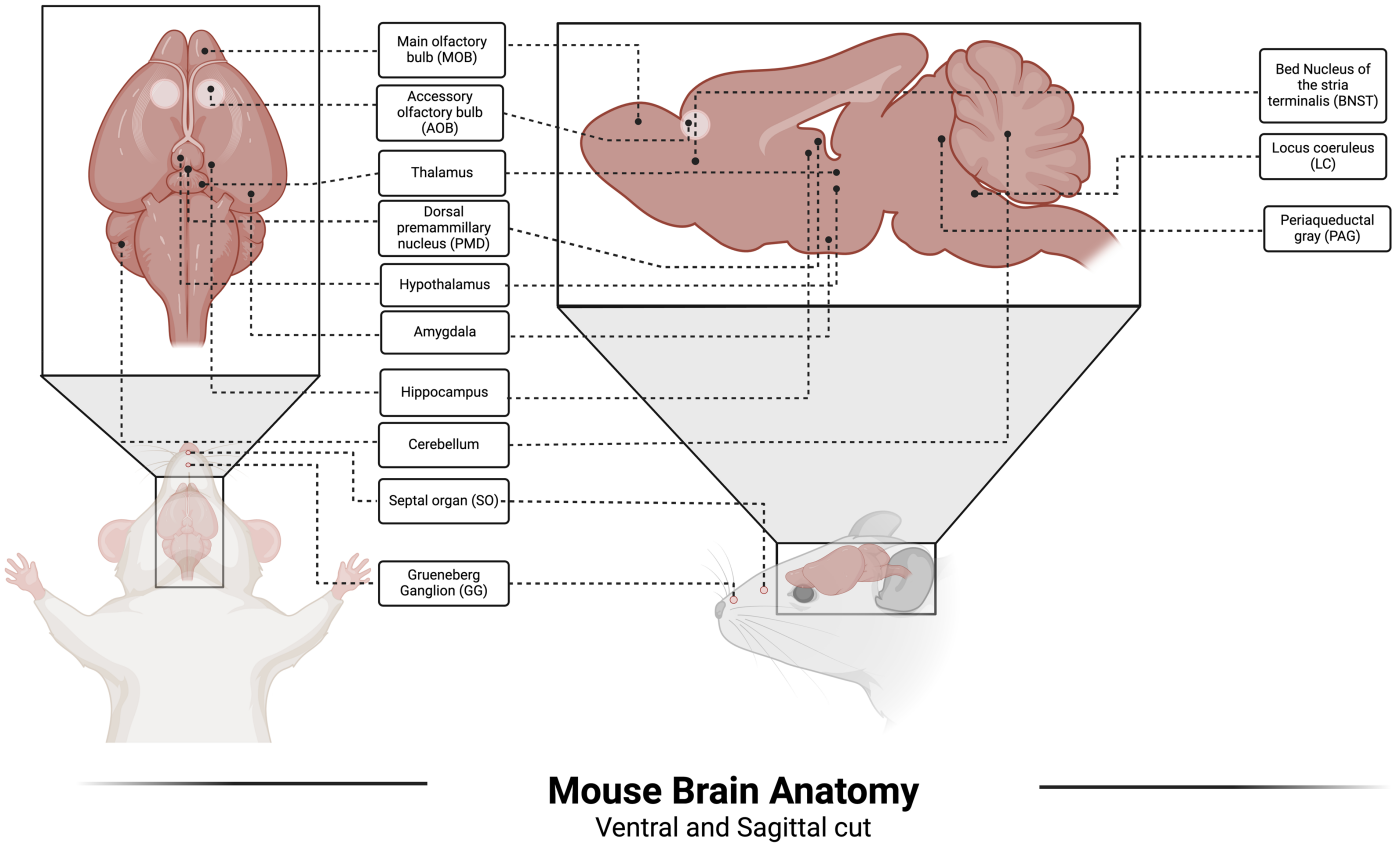
Mouse brain anatomy with olfactory organs and cerebral areas.

The GG was identified in 1973 (Grüneberg, 1973), but its involvement in rodent olfaction has only been established during the past two decades. Fuss et al. (2005) documented the expression of olfactory marker protein (OMP) in GG cells and their projection to the necklace glomeruli of the MOB. This finding was supported by Zimmerman and Munger (2021) and Koos and Fraser (2005). Approximately 15% of the OMP-positive GG cells in neonate rodents express distinct TAAR subtypes (Fleischer et al., 2007), and as stated earlier, TAARs are highly associated with the detection of amine compounds found in the urine of various rodent predators. Of note, Brechbühl et al. (2013) found activation of GG neurons by kairomones (2,4,5-trimethylthiazoline and 2-propylthietane), and that detection of alarm pheromones also activated GG (Brechbühl et al., 2008). In addition, 2-propylthietane (stoat kairomone) has been shown to activate GG (Perez-Gomez et al., 2015). However, evidence of the role of GG in the detection of other kairomones is limited. Fleischer (2021) also suggested investigating the connectivity between the GG and fear- and stress-associated cerebral centers to understand the neuronal circuitry. Although evidence has accumulated for the role of the GG in the detection of TMT and other kairomones, the evaluation of cat odor on the GG is yet to be performed. Brain anatomy with olfactory organs involved in kairomone signaling is depicted in Figure 2.

### Synergistic action of dual organs

Inactivation (temporary) of the amygdala olfactory cortex (AOC) in rats using local microinjections of GABA_A_ agonist (muscimol) reduced the fear behavior from exposure to fox urine, suggesting a role for AOC in kairomone signaling. Since the AOC interconnects the MOS and accessory olfactory system (AOS), the involvement of both systems has been suggested in kairomone signaling (Wernecke and Fendt, 2015). Similarly, the sensory neurons present in the dorsal and ventral olfactory epithelium were activated by phenylethylamine, implying the importance of both the MOS and GG in the detection of kairomones (Ferrero et al., 2011). Taken together, MOS, AOS, and GG have been implicated in kairomone signaling. In line with this, Takahashi and McGowan (2014) also provided evidence for the overlapping functions of different olfactory systems in rodents for predator odors and suggested a need for a detailed investigation of the neural circuits of kairomone signaling.

The detection of estrus by male mice involves the synergistic action of the MOS and VNO (Achiraman et al., 2010), as estrus detection is important for the mutual benefit of both males and females. Similarly, the detection of kairomones is crucial for determining the survival of both sexes of rodents. Therefore, it is functionally important that rodents adopt a sophisticated mechanism through the synergistic action of multiple organs (MOS/VNO, VNO/GG, GG/MOS, or all three). Nevertheless, the role of the septal organ (SO) in kairomone signaling has not been studied in rodents. Taken together, it is crucial to study the responses of all potential kairomones at various olfactory system and subsystem levels (MOS, VNO, and GG) to elucidate the molecular underpinnings of the complex signaling of kairomones and synergistic mechanisms.

## Cerebral areas involved in kairomone signaling

### Amygdala, the bed nucleus of stria terminalis and locus coeruleus

Various structural components, such as the amygdala, hypothalamus, hippocampus, and bed nucleus of the stria terminalis (BNST), are pivotal in processing kairomone stimuli. The BNST receives signal integration from the amygdala, hypothalamus, and hippocampus (Canteras, 2002).

The amygdala in laboratory rats was activated when exposed to TMT (Muller and Fendt, 2006). The amygdala circuitry contains many interconnected nuclei that connect various interneurons and facilitates the expression of specific behaviors associated with aversive cues in rodents (Janak and Tye, 2015). This, perhaps, suggests a role for nuclei of the amygdala in kairomone signaling. It is known that the amygdala consists of three well-defined sub-nuclei, in which the central nucleus of the amygdala (CeA) regulates fear responses (Ressler, 2010). It was evidenced that electrical stimulation of CeA resulted in conditioned fear responses, whereas lesions in the amygdala prevented the expression of such behaviors (Davis, 2000). C-fos expression was observed in the hypothalamic paraventricular nucleus (PVN) upon cat exposure, implying a role for PVN in kairomone signaling (McGregor et al., 2004). In addition, the medial amygdala (MeA) expressed two-to-five-fold increases in c-fos upon exposure to various predator odors such as recombinant Feld4, cat fur odor, 2-phenylethylamine, and 2-propylthietane, which proved the involvement of MeA in kairomone signaling. It was also evidenced that the MeA and ventromedial nucleus of the hypothalamus (VMH) are converging areas for 2-propylthietane signaling (Perez-Gomez et al., 2015). Similarly, MeA, basomedial amygdala, and BNST have been reported to provide major inputs to the defensive system in the brain, suggesting the possible role of these areas in kairomone signaling (Canteras, 2002). In contrast, however, others suggest that the CeA has minimal or no role in sensing TMT and cat odors (Takahashi et al., 2005; Martinez et al., 2011). Liu et al. (2021) documented that neurons of the basolateral amygdala (BLA) mediate the fear behavior in rats by increasing heart rate and freezing. However, the BLA and MeA were activated by cat odor exposure (Takahashi et al., 2007). Similarly, Bindi et al. (2018) found activation of the BLA nucleus in rodents exposed to live cats. We infer that the activation of different brain areas was due to the odor chemistry and individuality of the predator. We also suggest that the responses in rodents may not be well differentiated in the cerebral areas for different kairomones, unlike the differential primary reception of kairomones, which occurs in different sensory systems or subsystems. In addition, given the evidence of amygdala activation during live cat exposure, it is likely that visual signals could also serve as synergistic stimuli with olfactory stimuli, as observed in gilt responses to boar exposure (Signoret, 1971).

The BNST processes information and responds to threats through its well-connected network with other brain areas (Lebow and Chen, 2016). For example, the MeA is connected to the posterior BNST and is involved in sex differentiation. Also, the presence of estrogen receptors (alpha and beta) and androgen receptors in the anterolateral BNST imply that the regulatory mechanisms of BNST are sexually dimorphic for sustained fear and anxiety (Lebow and Chen, 2016). Stress-induced danger signals cause corticosterone-releasing factor (CRF) expression in rodents (Lucas et al., 2013), which may subsequently signal BNST. Of note, BNST has control over various neurotransmitters linked to CRF signaling and regulates social, anxiety-like, depression and feeding behaviors (Young and Tong, 2021). The BNST is also interconnected with the hypothalamus, hippocampus, and amygdala which, in coordination with other areas of the brain, controls various behavioral responses to emotion and stress (Hammack et al., 2021). Above all, c-fos expression in the BNST following predator odor exposure attested to the crucial role of the BNST in mediating kairomone-associated effects (Butler et al., 2016). The locus coeruleus (LC) has terminal projections on the BNST, and the way in which the LC projects to the BNST raises the possibility of a connection between BNST-modulated arousal, attention, and cognitive flexibility. The LC also responds to stress through CRF (Poe et al., 2020). As attested by hallmark reviews, the LC is a key component that releases noradrenergic neurotransmitters and mediates fight-or-flight responses to kairomone signaling (Charmandari et al., 2015; Morris et al., 2020). This indicates that the involvement of stress associated physiological responses in kairomone signaling is mediated by the LC. Taken together, both BNST and LC have proven roles in kairomone signaling.

Kairomone signaling in the LC and BNST, in part, influences their sexual dimorphism. However, the level at which complete establishment of sexual dimorphism occurs in kairomone signaling remains an open question. Since the BNST is connected to all major cerebral areas, it is prudent to investigate the link between the BNST and sexual dimorphism across different developmental stages in rodents.

### Dorsal premammillary nucleus (PMd) and periaqueductal gray (PAG) and hypothalamus

Convincing reports are available on the role of PMd in mediating fear-associated neuronal effects. The PMd receives inputs from the anterior hypothalamus, which integrates multiple inputs from various regions (cortex, amygdala, and hippocampus) (Canteras and Swanson, 1992). Lesions in the PMd reduced risk assessment behaviors toward live cats (Markham et al., 2004). This was corroborated by Dielenberg et al. (2004), who found c-fos expression in the PAG of rats upon exposure to cat odor. Taken together, the PMd has been shown to be activated during innate fear responses towards cat odor and live cats. However, the roles of the PMd and PAG in detection of fox odor, TMT, and odors from other related predators, have yet to be elucidated.

**Figure 3:**
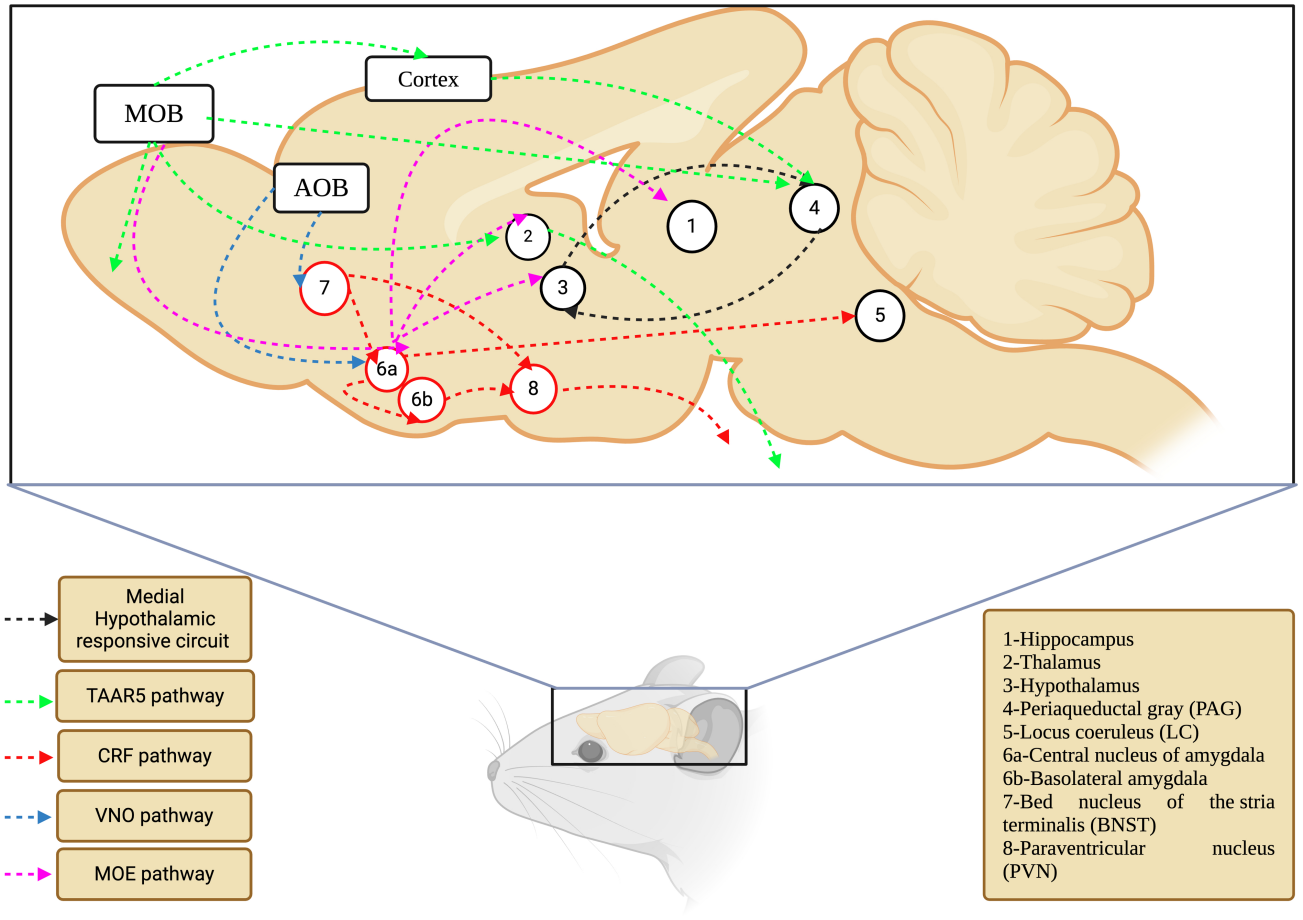
The olfactory network of rodents that mediate kairomone signaling. Each color depicts separate pathway which are converging different locations in the coordination of the signaling events (TAAR5-Trace Amine Associated Receptor 5; CRF-Corticotropin Releasing Factor; VNO-Vomeronasal Organ; MOE-Main Olfactory Epithelium).

The hypothalamus is an intermediate but essential component of neuronal signaling in response to various chemical signals, and it receives signals from many regions, including the amygdala (Davis et al., 2010). Ultimately, various cells in the hypothalamus secrete hormones that act on the pituitary gland and regulate the endocrine system (Clarke, 2015). The action of the endocrine system depends on the signals conveyed by the chemical compounds, wherein sex pheromones activate the hypothalamic–pituitary-gonadal (HPG) axis, and danger signals activate the hypothalamus-pituitary–adrenal (HPA) axis. In this sense, c-fos expression was observed in three medial hypothalamic nuclei, including the ventromedial hypothalamus, of rodents exposed to cats (Motta et al., 2009). A similar pattern has been observed for TMT through electrolytic and neurotoxic lesions induced in the anterior and ventromedial hypothalamus (Pagani and Rosen, 2009). It is important to note that the ventromedial nucleus of the hypothalamus has axonal projections from the amygdala (Yamamoto et al., 2018), which is a key organ in responding to fear stimuli, as explained earlier in this review. The behavioral and neuronal effects of various kairomones studied/verified in rodents are listed in Table 3. The kairomone-signaling brain maps and their respective pathways in rodents are shown in Figure 3.

**Table 3:** Neuronal effects of various kairomones tested with different species and strains of rodents.

Test animals	Compound/kairomone sources used	Behaviors tested	Neuronal evidence	Reference
Australian Albino Wistar rats	Trimethylthiazoline	Grooming and escape behaviors	C-Fos in the accessory olfactory bulb and its projection areas, the main olfactory bulb and central and cortical amygdala;	Staples et al. (2008a)
Male Sprague-Dawley rats		Fear-related behaviors	The bed nucleus of the stria terminalis (BNST)	Fendt et al. (2005)
Male Sprague-Dawley rats	2,4,5 dihydro 2,5 trimethylthiazoline	Freezing behavior	Olfactory system and trigeminal nerve system	Ayers et al. (2013)
Naïve wild-type male mice (C57BL/6)	2,5-dihydro- 2,4,5-trimethylthiazole with other compounds	Avoidance, defensive behaviors, and risk assessment episodes.	C-Fos expression in AOB, and Ca2^+^-expression in anterior and posterior AOB; necklace system; C-Fos positive cells in the immediate vicinity of PDE2A+ (phosphodiesterase 2A) glomeruli; c-Fos+ cells in the medial amygdala.	Perez-Gomez et al. (2015)
Adult C57BL/6 mice offspring	2,3,5-Trimethyl-3-thiazolin	Foraging activity, stress-related behavior, locomotor activity, frequency of visits, risk assessment and escape behavior	Paraventricular nucleus of the hypothalamus.	St-Cyr et al. (2018)
Inbred C57BL/6J mice.	Feld4	Avoidance behavior, risk assessment behavior	Vomeronasal sensory neurons; c-Fos response in the anterior AOB, posterior AOB	Papes et al. (2010)
Male Wistar rats	Cat collar	Stimulus contact, grooming and rearing	Fos expression in medial amygdala and bed nucleus of the stria terminalis, prelimbic cortex, lateral septum, lateral and medial preoptic areas, and dorsal premammillary nucleus, ventromedial hypothalamic nucleus, paraventricular nucleus of the hypothalamus, periaqueductal gray, and cuneiform nucleus.	McGregor et al. (2004)
OMP-GFP mice	Urine of mountain lion and raccoon; Urine and anal gland secretions from the skunk	Walking distance index and immobility index	GG neurons	Lopes et al. (2022)
Naive male Sprague-Dawley rats	Fox urine	Fear behaviors (locomotor activity and avoidance behavior)	Amygdalar olfactory cortex	Wernecke et al. (2015)

Overall, the neuronal pathways of kairomone signaling were assigned to possible levels, as evidenced by the sequential connection between the five key areas (amygdala, hypothalamus, BNST, PMd, and PAG), relative expression of c-*fos* and other genes, and pronounced endocrine changes. However, a holistic approach that includes all five areas must be considered to test for any putative or novel kairomone-odor/compound. Furthermore, shedding light on the primary sensory organ for kairomone compounds (MOS/AOB/GG) is also essential. Together, this would pave the way for understanding the perception and neuronal signaling of kairomones in rodents.

## Bouquet hypothesis

It is possible, even likely, that kairomones are odor complexes and will activate multiple cerebral areas (bouquet hypothesis). Indeed, Apfelbach et al. (2015) suggested a mixture of compounds can deliver more relevant information to the receiver than a single compound, similar to the effect of signature mixtures and pheromones (Wyatt, 2010). It was also highlighted that the molecules in the signature mixtures, irrespective of their size, are perceived by the main and/ or accessory olfactory systems. The key fact is that the signature mixtures were proposed as a receiver-side phenomenon that helped them to distinguish each other (Wyatt, 2010). By taking support from these inferences, we propose the kairomone mixture can also be considered as a signature mixture at the receiver level (specifically, rodents). It is possible that the signature mixtures of kairomones can contain more than one compound, and each compound could mediate a subset of functions in the prey at the behavioral, physiological, and neurosystem levels. We also take the support for bouquet hypothesis of kairomones from Brechbühl et al. (2015), who evidenced that when presented to rodents, lion urine increased the blood pressure and heart rate and caused neuronal changes in the GG, implying that the mixture of compounds in the urine has a high potential to act as kairomones. However, a bouquet hypothesis for kairomones requires further investigation and should be explored by comparing the source vs. compound(s) in the same behavioral paradigm.

## Utilizing advanced technologies for kairomone studies

Optogenetics enables precise manipulation of specific neurons using light, allowing researchers to examine neural circuits with exceptional temporal precision. Deisseroth (2015) illustrated the application of optogenetic tools to modulate neural activity, uncovering the roles of various brain regions and pathways in behavioral reactions to predator odors. Similarly, chemogenetics offers a different strategy by employing engineered receptors and custom drugs to selectively activate or inhibit particular neurons. Roth (2016) reviewed the use of DREADDs (Designer Receptors Exclusively Activated by Designer Drugs) in neuroscience, emphasizing their effectiveness in investigating complex behaviors and neural networks. Optical imaging technologies, such as genetically encoded calcium indicators, have transformed the real-time visualization of neuronal activity. In this regard, Tian et al. (2012) detailed the creation and use of these indicators, which facilitate the observation of dynamic brain processes in response to different stimuli, including predator odors.

Virus tracing techniques have enhanced our understanding of neural connectivity and the circuits involved in specific behaviors. Zingg et al. (2017) used adeno-associated virus (AAV)-mediated anterograde transsynaptic tracer tagging coupled with tracer-dependent transgene expression to map neural pathways associated with defense behaviors. It shed light on how various neuron subpopulation in the brain regions interact in response to particular stimuli. Despite the availability of these advanced techniques and approaches, not all predator odors/kairomones have been investigated using these approaches. Ultimately, utilization of these state-of-the art techniques would help understand the neuronal effect of explored and unexplored kairomones.

## Concluding remarks

It is evident from the above reports that many variables are associated with kairomone testing in rodents. Behavioral differences between rodent species are evident, such as rats and mice differing in their behavior when exposed to the same kairomones. In the wild, urine, urinated matrices, and feces are prone to microbial degradation. However, the fur of predators may not be decomposed, as are other body secretions and, therefore, may carry viable information to rodents. This could explain why kairomones identified in fur elicited a heightened long-term response in rodents. However, both metabolized and non-metabolized original compounds are crucial for the detection of predators by rodents. These compounds need to be tested by comparing fresh and aged kairomone sources in behavioral assays, neuronal studies, and, most importantly, in chemical analysis. It has been suggested that cats carry kairomones mainly in their fur, and that ferrets release TMT as one of their major kairomones. However, the presence of TMT in fox feces has not been confirmed in all studies and requires further investigation. Nevertheless, the chemical identities of the odor sources of many predators remain unexplored but have the potential to be investigated beyond what was initially imagined. At the neuronal level, the activation of various cerebral areas depends on many variables, including the nature of the predator, Kairomone source/compound, and exposure conditions. The type of secretion also contributes to variations in the neuronal effects. Therefore, a comparative analysis of kairomones and their respective kairomone-olfactory brain maps would help to understand the mechanisms in a comprehensive way and foster further research.
